# Transmitted/Founder Simian Immunodeficiency Virus Envelope Sequences in Vesicular Stomatitis and Semliki Forest Virus Vector Immunized Rhesus Macaques

**DOI:** 10.1371/journal.pone.0109678

**Published:** 2014-10-31

**Authors:** Ratish Gambhira, Brandon F. Keele, John B. Schell, Meredith J. Hunter, Jason P. Dufour, David C. Montefiori, Haili Tang, John K. Rose, Nina Rose, Preston A. Marx

**Affiliations:** 1 Division of Microbiology, Tulane National Primate Research Center, Tulane University, Covington, Louisiana, United States of America; 2 AIDS and Cancer Virus Program, SAIC-Frederick Inc., Frederick National Laboratory for Cancer Research, Frederick, Maryland, United States of America; 3 Department of Pathology, Yale University, New Haven, Connecticut, United States of America; 4 Department of Medicine, Duke University Medical Center, Durham, North Carolina, United States of America; University of Pittsburgh Center for Vaccine Research, United States of America

## Abstract

Identification of transmitted/founder simian immunodeficiency virus (SIV) envelope sequences responsible for infection may prove critical for understanding HIV/SIV mucosal transmission. We used single genome amplification and phylogenetic analyses to characterize transmitted/founder SIVs both in the inoculum and in immunized-infected rhesus monkeys. Single genome amplification of the SIVsmE660 inoculum revealed a maximum diversity of 1.4%. We also noted that the consensus sequence of the challenge stock differed from the vaccine construct in 10 amino acids including 3 changes in the V4 loop. Viral env was prepared from rhesus plasma in 3 groups of 6 immunized with vesicular stomatitis virus (VSV) vectors and boosted with Semliki forest virus (SFV) replicons expressing (a) SIVsmE660 gag-env (b) SIVsmE660 gag-env plus rhesus GM-CSF and (c) control influenza hemagglutinin protein. Macaques were immunized twice with VSV-vectors and once with SFV vector and challenged intrarectally with 4000 TCID_50_. Single genome amplification characterized the infections of 2 unprotected animals in the gag-env immunized group, both of which had reduced acute plasma viral loads that ended as transient infections indicating partial immune control. Four of 6 rhesus were infected in the gag-env + GM-CSF group which demonstrated that GM-CSF abrogated protection. All 6 animals from the control group were infected having high plasma viral loads. We obtained 246 full-length envelope sequences from SIVsmE660 infected macaques at the peak of infection and determined the number of transmitted/founder variants per animal. Our analysis found that 2 of 2 gag-env vaccinated but infected macaques exhibited single but distinct virus envelope lineages whereas rhesus vaccinated with gag-env-GM-CSF or HA control exhibited both single and multiple env lineages. Because there were only 2 infected animals in the gag-env vaccinated rhesus compared to 10 infected rhesus in the other 2 groups, the significance of finding single env variants in the gag-env vaccinated group could not be established.

## Introduction

Understanding the early events of HIV infection may play an important role in controlling transmission especially through immunization [1]–[7]. Characterization of transmitted/founder (T/F) viral envelope sequences at the peak of viral infection provides the exact nucleotide sequences of individual viruses responsible for establishing early clinical infection after breaching the mucosal barrier. Although several methods have been proposed previously for characterizing the virus responsible for transmission [8]–[10], they were limited by mutations caused by *Taq* polymerase and cloning bias. However, the single genome amplification (SGA) method has enabled extensive characterization of the T/F viruses in HIV and SIV infections [10], [11]–[18]. The SGA technique surpasses previous PCR based techniques in accurately representing the proportion of viral variants in a population as well as preventing any *Taq* induced errors including single nucleotide polymorphisms, Indels and in vitro recombinations [19], [20].

SIV infection of rhesus macaques (RhMs) is the generally accepted model for testing HIV vaccine strategies [21]. The envelopes of both SIV and HIV are heavily glycosylated and almost 40% of their mass is derived from carbohydrates [22], [23]. After SIV infection of macaques, the envelope evolves rapidly in the variable region 1 and 4 (V1 and V4) resulting in significant changes in the glycosylation patterns [24], [25]. Understanding the molecular evolution of the envelope should lead to developing a better vaccine that could prevent SIV/HIV transmission. In this study, we characterized the SIVe660 inoculum full-length envelope sequences and compared them with the vaccine construct. Secondly, we evaluated the number of T/F viruses in infected-SIV-immunized macaques having transient viral loads compared to those that exhibited high and sustained plasma virus loads (PVLs). Additionally, we identified the variations in the envelope sequences between the inoculum and the T/F virus sequences. To achieve these aims, we performed SGA on the stock inoculum and plasma from VSV immunized and SIVsmE660 challenged macaques [26], [27]. The vaccinated groups included macaques immunized with VSV expressing gag and env derived from SIVsmE660 (Group I); SIVsmE660 gag, env along with rhesus GM-CSF (Group II); and VSV expressing Influenza HA/control, group (Group III). Our results demonstrate that the transiently infected macaques from the gag-env group exhibited only one founder virus as compared to both multiple founder viruses and single env variants found in the macaques immunized with gag-env-GM-CSF and HA/control groups.

## Materials and Methods

### Animals and Vaccinations

All animals were born and raised at the Tulane National Primate Research Center (TNPRC). All procedures were approved by the TNPRC Institutional Animal Care and Use Committee and were in accordance with the *Guide for the Care and Use of Laboratory Animals* and the *Animal Welfare Act*. The TNPRC maintains an AAALAC-I accredited animal care and use program. All animals are maintained on a 12 hour light/dark cycle and fed a commercially available nonhuman primate chow twice daily and water is available ad libitum. All nonhuman primate animal rooms are maintained at a temperature of 64–84°F with a humidity range of 30–70%. All nonhuman primate rooms are provided 10–15 fresh air changes per hour. All animals receive enrichment as part of the TNPRC Policy on Environmental Enrichment. Enrichment includes durable and destructible objects in their cage, perches, various food supplements (fruit, vegetables, treats) foraging or task-oriented feeding methods fed a minimum of three times weekly, and human interaction with caretakers. All animals are observed twice daily by care staff and a veterinarian is notified if abnormalities are present. All procedures were conducted with the use of ketamine hydrochloride (10 mg/kg intramuscularly; Ketaset, Fort Dodge Laboratories). Animals are administered buprenorphine hydrochloride (0.03 mg/kg intramuscularly twice daily; Buprenex, Reckitt & Colman) for any procedures expected to induce pain or distress. All animals were released to the TNPRC colony upon completion of the study. Indian-origin rhesus macaques were primed with either VSV expressing (1) E660 gag, env, (2) VSV carrying E660 gag and env along with GM-CSF or (3) VSV vectors expressing the A/Vietnam/1203/2004 H5 hemagglutinin (HA) from influenza. The detailed vector design and vaccination schedules are published [27] and briefly depicted here in the Table 1 for clarity. For the VSV prime and first boost a total of 10^8^ PFU of both VSV-E660 gag and VSV-E660 envG viruses were delivered intramuscularly (i.m.; 6×10^7^ PFU) and intranasally (i.n.; 4×10^7^ PFU) to each monkey. The second boost used Semliki Forest virus (SFV) replicon (SFVG) packaged by a VSV glycoprotein (G) particle boost and was delivered by intramuscular injection only. Each animal received 1×10^6^ infectious units (i.u.) of both SFVG-gag and SFVG-envG. Animals in the control group were primed and boosted with VSV vectors expressing influenza HA protein. The E660 *env* and *gag* genes were obtained by amplifying these genes by PCR from DNA of SIVsmE660 infected CEMx174 cells.

**Table 1:** Immunization and Challenge Schedule of Rhesus Macaques.

Immunized Macaque Groups	Total Envelopes Sequenced	Number of T/F viruses
VSV-Env-Gag		
(Group I)		
DF38	21	1
DG21	19	1
VSV-Gag-Env-GM-CSF		
(Group II)		
EA28	21	1
EF23	18	1
EF28	22	3
EJ74	26	6
VSV-HA		
(Group III)		
FT80	22	1
N288	16	1
DD04	15	1
FG05	15	3
EN82	17	3
EP84	23	5

*6 adult rhesus macaques in each group; i.m  =  intra-muscular; i.n  =  intra-nasal; i.r  =  intra-rectal.

NJ  =  New Jersey strain; I =  Indiana strain.

### Plasma viral load determination

The PVLs were determined by Branched DNA (bDNA) analysis of serial plasma samples (Siemens Diagnostic Clinical Laboratory, Berkeley, CA). All the samples were assayed in duplicate. The lower limit of detection was 160 copies/ml.

### SIVsmE660 challenge of rhesus macaques

All animals in the study were intrarectally (IR) inoculated with 4000TCID_50_ of SIVsmE660. The virus stock was kindly provided by Dr. Philip R Johnson (University of Pennsylvania, Phliadelphia, PA).

### Single Genome Amplification (SGA)

SGA was performed to characterize envelope sequences of the SIVsmE660 challenge stock and infected animals as described previously [11]. In brief, the viral RNA (vRNA) was extracted from inoculum stock and the infected macaque plasma samples using the QIAamp viral RNA isolation kit (Qiagen, Valencia, CA). Viral particles were concentrated by centrifuging at 23,600×g at 4°C if the viral copy number was too low or diluted with PBS if the number was too high to obtain approximately 40,000 vRNA copies per extraction. The first cDNA strand was synthesized from viral RNA with SIVsmE660 R1 primer using the cDNA synthesis kit (Life Technologies, Grand Island, NY). The cDNA was synthesized by keeping the mixture at 50°C for 1 hr followed by another hour of incubation at 55°C. The cDNA was serially diluted and subjected for 2 rounds of nested PCR using SIVsmE660 envelope specific primers. 3–5 ul of the PCR amplified product was checked on agarose gel and the dilution was selected where <30% of the amplicons were positive. The PCRs were repeated at the selected dilution to obtain 20–30 full-length envelope amplicons originating from single templates. All the amplicons were directly sequenced using Sanger sequencing with 8 overlapping primers.

### Phylogenetic analysis of full-length env sequences

All the contiguous sequences were initially analyzed using Sequencher (Gene Codes, Ann Arbor, MI) for nucleotide discrepancies. Further the full-length envelope sequences were aligned using the JALVIEW program (http://www.jalview.org/) and Neighbor-Joining phylogenetic trees were constructed using FigTree (http://tree.bio.ed.ac.uk/software/figtree/), Dendroscope (http://ab.inf.uni-tuebingen.de/software/dendroscope/), JALVIEW and Phylogeny.fr (http://phylogeny.lirmm.fr) programs. We also used various tools (Highlighter plot, Poisson-Fitter analysis, Sequence publish) from Los Alamos National laboratory web site for analyzing the T/F viral envelope variants (http://www.hiv.lanl.gov).

## Results and Discussion

### Kinetics of viral loads in VSV vaccinated–breakthrough virus infected macaques

**Figure 1:**
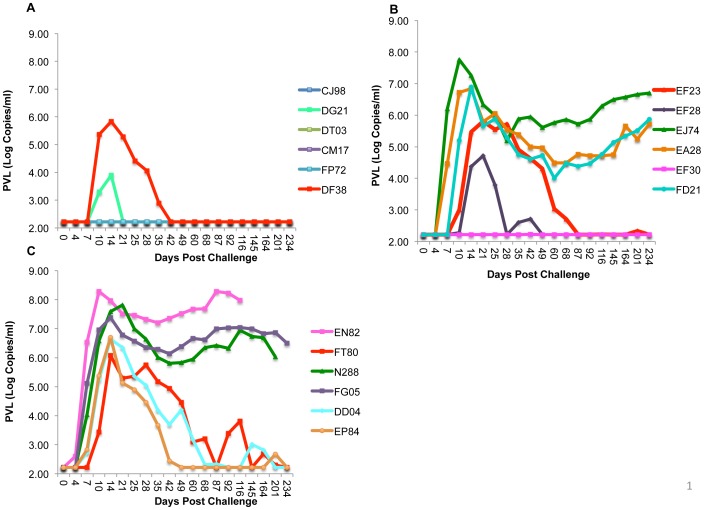
Plasma virus loads (PVLs) in immunized and control rhesus macaques challenged with SIVsmE660. The PVLs were assayed from serial plasma samples collected from rhesus vaccinated with vesicular stomatitis-semliki forest virus vectors and challenged with SIVsmE660. The graphs depict the PVLs in Log_10_ scale. (A) In SIVe660 gag-env immunized group (Group I). (B) SIVe660 gag-env immunized plus GM-CSF group (Group II) and (C) the Influenza HA control group (Group III).

In total, 3 groups of 18 rhesus macaques were IR challenged with 4000 TCID_50_ SIVsmE660. In gag-env immunized macaques, 2 of 6 animals had transient viral loads above the baseline level (DG21 and DF38). Both rhesus had reduced peak viral loads at day 14 post-challenge 10^3.9^ to 10^5.8^ compared to the control group (Fig. 1). The plasma viral loads were undetectable by day 21 in DG21 and by day 42 in DF38 (Fig. 1A). The remaining four macaques, in the gag-env group had undetectable viral loads throughout the study period. Importantly, after the depletion of CD8^+^ T cells, both transiently infected macaques (DF38, DF21) but not the 4 completely protected macaques exhibited rebound in viral loads confirming low-level systemic infection. The Gag-env-GM-CSF group immunized animals had 5 of 6 rhesus infected, with peak PVLs ranging from 10^4.7^ to 10^7.8 on^ days 10 to 21 post-infection (Fig. 1B). All HA-vaccinated-control macaques were infected and had the highest peaks (10^6.0^ to 10^8.3^) (Fig. 1C. While some reached peak by days 10 (EN82) or 14 (FG05, DD04, EP84, FT80) others showed peak viral loads by day 21 (N288) (Fig. 1C). PVLs of the efficacy experiment (Fig. 1) were published [27] and are also shown here for clarity.

VSV/SFV vector immunization resulted in the production of high levels of antibody to SIVsmE660 [27]. SIVsmE660 is more susceptible to antibody neutralization, as compared to SIVmac239 or SIVmac251 [28] thus providing the rationale for using SIVsmE660 virus. Further, gag was included in the vaccine vectors because previous reports have suggested that both antibody and T cell responses play a key role in SIV control [29]–[31]. The challenge dose of 4000 TCID_50_ by IR route was determined based on the previous studies [32].

A major concern in using SIVsmE660 as a challenge virus was its susceptibility to TRIM5α restriction. Only TRIM5 α ^TFP/CypA^ allele showed a significant effect on acquisition of SIVsmE660 after repeated IR challenges [33], [34]. None of the animals in this study had the controlling genootype.

### SGA analysis of the SIVsmE660 challenge stock

**Figure 2:**
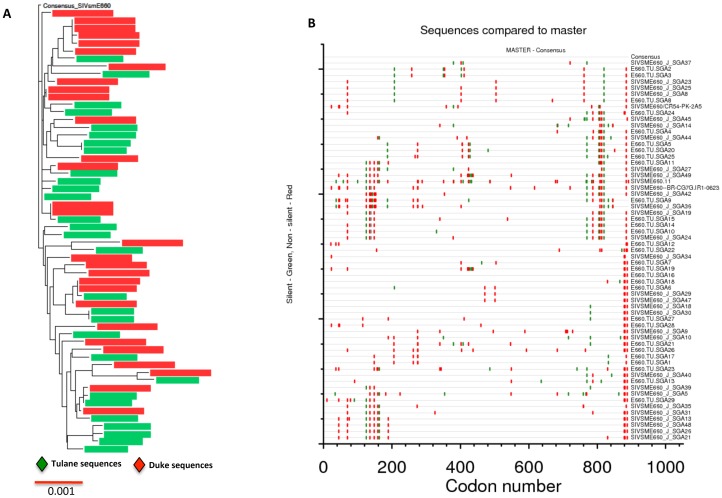
Phylogenetic analysis and highlighter plots of the SIVsmE660 challenge stock. A total of 58 full-length envelope sequences were analyzed from the SIVsmE660 stock viral swarm and were compared to the derived consensus E660 sequence for silent and non-silent mutations. (A) Neighbor-joining phylogenetic tree with all the 58 env sequences compared to consensus sequence derived from SIVsmE660 inoculum. (B) Highlighter plot showing the silent (green)/non-silent (red) mutations among different full-length envelope sequences as compared to the consensus SIVsmE660 sequence.

To determine the genetic diversity and the phylogenetic relationships among different envelope sequences, SGA was done on the stock in 2 laboratories. Twenty-nine full-length envelope sequences were obtained at the Tulane primate center (Fig. 2A, Tulane sequences in green) and 29 full-length envelope sequences from the same stock virus provided by Dr. David Montefiori's group from Duke University (Duke sequences in red). Sequence were aligned, analyzed and are shown in Fig. 2. Nucleotide sequences from both the institutions were interspersed in the phylogenetic tree indicating their close relationship of being from the same stock. The maximum overall env diversity for the stock was 1.4%. A consensus sequence was generated from inoculum envelope sequences and was compared to each sequence (Fig. 2A). Three out of fifty-eight envelope sequences had an insertion of proline, threonine and alanine (P, T, A) from 136–138 amino acid position of V1 loop. No major changes were seen in the rest of the variable loops of gp120. Several notable amino acid differences were seen in the gp41 cytoplasmic tail (I882V L885F and T888A) as compared to the SIVsmE660 consensus sequence (data not shown). Additional silent (synonymous) and amino acid altering mutations (non-synonymous) were identified in the stock envelope sequences as depicted in the highlighter plot (Fig. 2B). Taken together the viral inoculum contained diversified envelope sequences and these sequences will be deposited in the Genbank.

Previous studies in other laboratories showed that the SIVsmE660 diversity was 1.8%, which is comparable to SIVmac251 stocks (0.8–1.4% maximum envelope diversity) [35]. As indicated before 3 of 58 full-length envelope sequences showed a motif of proline, threonine and alanine (P, T, A) in the V1 loop (positions136–138). This exact mutation was observed in the SIVsmE660 stock virus that was previously characterized by SGA even though the significance of it is not yet clear [35]. Also, other than the variation mentioned in the V1 loop, the majority of other differences were limited to the cytoplasmic tail of gp41 between amino acids 880 to 890. The remaining envelope region is well conserved among different full-length envelope sequences derived from the SIVsmE660 viral swarm.

### Comparison of full-length stock envelope sequences to vaccine construct

**Figure 3:**
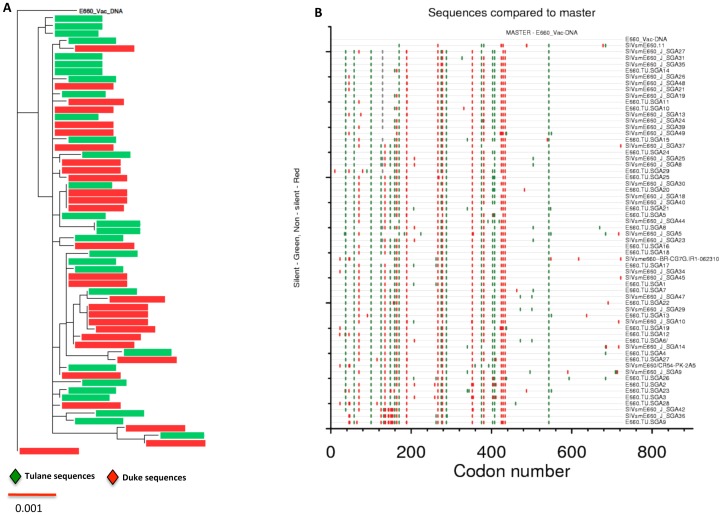
Comparison of E660 viral swarm sequences in the challenge stock to the E660 vaccine construct. The full-length E660 sequences were trimmed to match the vaccine construct sequence. (A) Neighbor-joining phylogenetic tree with all 58 env sequences compared against the vaccine construct. (B) Highlighter plot showing the silent (green) and non-silent (red) changes in all 58 trimmed envelope sequences compared to the E660-vaccine construct.

All full-length envelope sequences of SIVe660 inoculum were compared to the E660-envelope used in the vaccine construct (Fig. 3A). The stock viral sequences were trimmed to match the envelope sequence in the vaccine construct which lacked ∼500 bp at the C-terminus for facilitating the packaging of envelope into VSV vectors. The SIVsmE660 env nucleotide sequence used in the vaccine construct differed from the stock sequences on average by 2% and exhibited both silent and non-silent mutations (Fig. 3B). The inoculum consensus envelope sequence was also compared to the vaccine construct to determine the conserved and non-conserved changes between the two. In total, there were ten amino acid changes in the inoculum consensus compared to the vaccine construct. No major changes were noted in the variable loops of gp120 except for three amino acid changes in the V4 loop (G425S, E429 R and K434R) (data not shown). Finally, none of the swarm amino acid sequence was identical to the E660 vaccine construct sequence with the closest match being 11 amino acids apart.

### Identification of T/F viruses from gag-env (Group I) macaques

**Figure 4:**
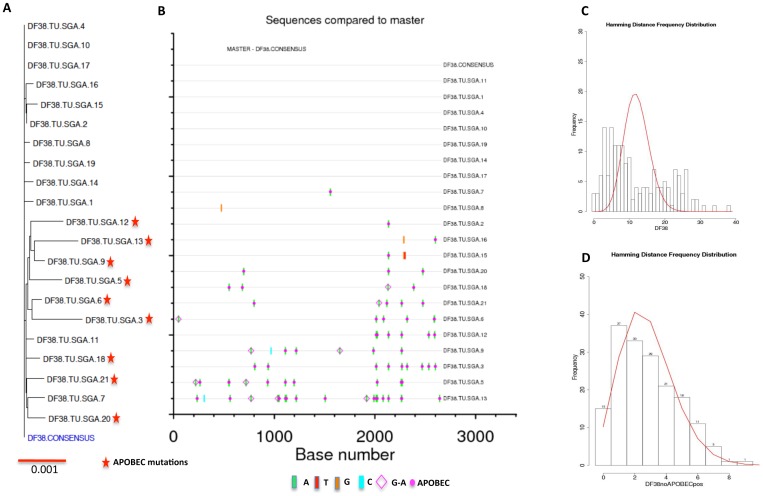
Phylogenetic and Poisson-Fitter analysis of DF38 macaque env sequences. In total 21 full-length env sequences were analyzed to identify the T/F virus from DF38. (A) Phylogenetic tree of DF38 full-length envelope sequences compared to derived DF38 consensus sequence. (B) Highlighter plot showing variations in nucleotides and APOBEC mutations in individual nucleotide sequences in comparison to DF38 consensus sequence. (C) Poisson-Fitter plots of DF38 env sequences including APOBEC mutations. (D) Poisson-Fitter plots of DF38 env sequences excluding the APOBEC mutations.

**Figure 5:**
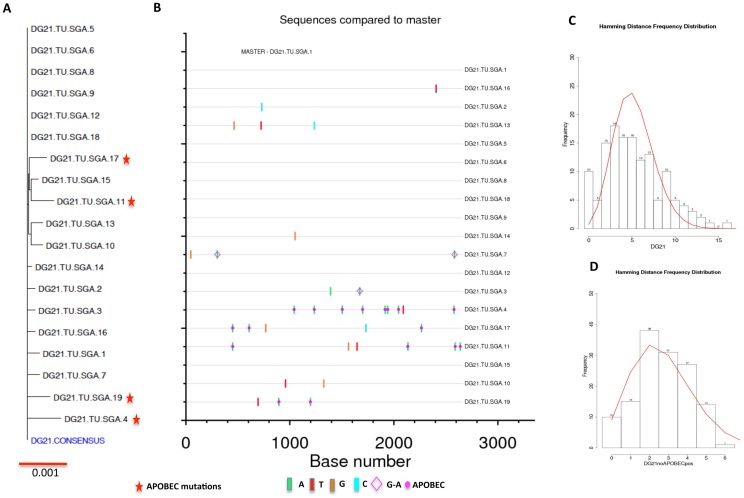
Phylogeny and Poisson-Fitter analysis of DG21 macaque env sequences. We analyzed 19 full-length envelope sequences from SIVsmE660 infected DG21 macaque. (A) phylogenetic comparison of DG21 env sequences to the generated consensus DG21 envelope sequence. (B) Highlighter plot exhibiting the nucleotide differences in DG21 env sequences compared to DG21 consensus sequence. (C) Poisson-Fitter plots of DG21 env sequences including APOBEC mutations. (D) Poisson-Fitter plots of DG21 env sequences excluding the APOBEC mutations.

In gag-env vaccinated animals, four out of six macaques exhibited apparent sterilizing immunity to the challenge, while the other two had transient viremia. SGA was performed on the day 14 (peak viremia) plasma samples from both macaques. We obtained in total 21 full-length envelope sequences from DF38 and the sequences were used to construct a phylogenetic tree. A consensus envelope sequence was generated and individual envelope nucleotide sequences were compared to the consensus. Highlighter plots were made to enumerate the number of T/F variants and identify the putative env sequence that initiated infection. (Fig. 4A). Nine out of twenty one (43%) sequences showed a significant number of APOBEC mutations (red star in Fig. 4A). These mutations were created by host restriction of the retrovirus and often obscure enumeration of T/F viruses from infected animals (Fig. 4B) [36]. After, accounting for these mutated sequences, a single low diversity variant was observed in DF38 representing infection with a single T/F variant. Of the remaining 12 sequences, 7 were identical to the consensus sequence and to themselves and represent the T/F sequence. Sequences that differed from consensus sequence contained only one or at most two mutations randomly distributed in the genome. Further, to confirm the infection with a single variant, we performed Poisson-Fitter analysis of SGA envelope sequences [37], which determines if frequency of mutations accumulate are consistent with Poisson distribution. This analysis distinguishes between the presence of one or more founder viruses from infected animals. The DF38 macaque putative founder virus fit the Poisson-Fitter analysis (after eliminating APOBEC mutations) confirming that this macaque was infected with only one founder virus (Fig. 4C and D). In case of DG21, the plasma viral load was low (<40,000 copies/ml) and hence we obtained viral RNA after pelleting the plasma sample as described in the methods. In total, 19 full-length envelope sequences were obtained and were analyzed phylogenetically (Fig. 5A) of which 4 sequences showed APOBEC mutations on the highlighter plot (Fig. 5B). Similar to DF38, after subjecting the sequences for Poisson-Fitter analysis this macaque was also infected with only one founder virus (Fig. 5C and D).

Additionally, we compared the consensus translated amino acid sequences of both DF38 and DF21 to consensus viral stock envelope sequence (data not shown). Both the sequences differed from consensus E660 by seven amino acids and each showed unique changes (DF38-T134S and DG21-V842A) as compared to consensus challenge stock.

In essence, the two infected rhesus in the gag-env immunized group (DF38 and DG21) had transient viral loads and both of the animals were infected with only one founder virus after eliminating the APOBEC mutations. In addition, when the CD8+T cells were depleted from these infected macaques, they showed rebound in viral loads indicating that these animals were infected but controlled the viral replication successfully upon vaccination. The remaining four macaques didn't reveal any viral loads even after CD8+ T cell depletion indicating that they exhibited a sterilizing immunity [27].

### Identification of T/F viruses from gag-env immunized plus GM-CSF (Group II)

**Figure 6:**
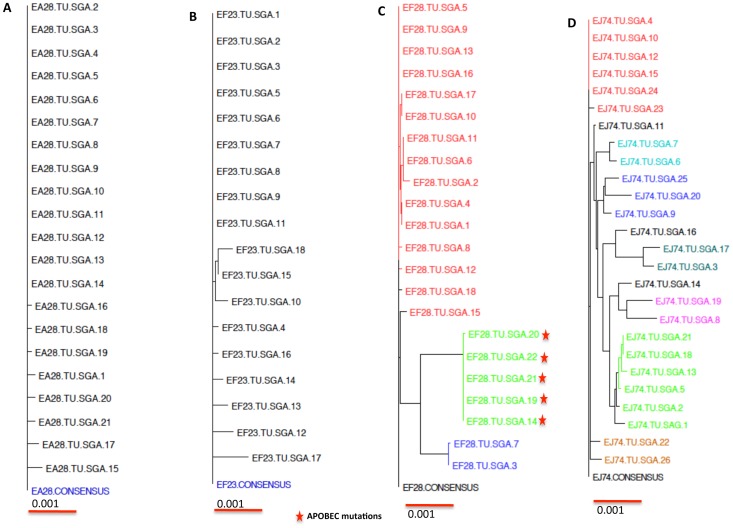
Phylogenetic analysis of full-length env sequences from Gag-Env-GM-CSF group (Group II)) macaques. All the macaques in this group were vaccinated and challenged with SIVsmE660. The full-length envelope sequences were compared to a derived consensus sequence from each animal (A) EA28 (B) EF23 (C) EF28 and (D) EJ74. Multiple lineages of T/F viruses infecting the same macaque were shown in different colors.

Following challenge with SIVsmE660, 5 of the 6 RhMs were infected in gag-env-GM-CSF group with relatively high PVLs that were comparable to the control group [Fig. 1C]. The results demonstrated that VSV-vectored GM-CSF abrogated the protective effect of the vaccine, since 4 of 6 animals were protected in gag-env group, whereas addition of GM-CSF reduced protection to 1 of 6 animals in gag-env-GM-CSF group as previously reported [26]. We performed SGA analysis on 4 of 5 infected animals at the peak of their viremia. After repeated attempts only partial sequences were obtained from the 5^th^ animal FD21, so this animal could not be analized. All the full-length envelope sequences were compared phylogenetically including the derived consensus sequence. The major lineages of different founder viruses are highlighted in various colors on the phylogenetic tree (Fig. 6). We obtained 21 full-length envelope sequences from EA28 and found only 1 transmitted virus (Fig. 6A). We analyzed 18 full-length envelope sequences from EF23 and noted only 1 founder virus (Fig. 6B). However, we found 3 different founder viruses, after analyzing 22 full-length Envelope sequences from EF28 (Fig. 6C) and 6 variants in 26 full-length envelope sequences from EJ74. There were a few other envelope sequences that were either recombinants or independent founder viruses but couldn't be categorized appropriately due to their limited number. None of the animals in this group showed APOBEC mutations (except 5 sequences from EF28). We also performed Poisson-Fitter analysis on the full-length envelope sequences obtained from various infected macaques (data not shown). The results demonstrate that EA28 and EF23 fit the Poisson-Fitter analysis, since both the samples were infected with only 1 founder virus and it did follow the star like phylogeny of evolution. However, both EF28 and EJ74 failed to fit this analysis, since they were infected with more than one transmitted viruses. Therefore, in this group two macaques (EF28, EJ74) were infected with multiple founder viruses and the other two (EA28, EF23) were infected with only one founder virus.

### Characterization of transmitted viruses from HA/control (Group III) rhesus macaques

**Figure 7:**
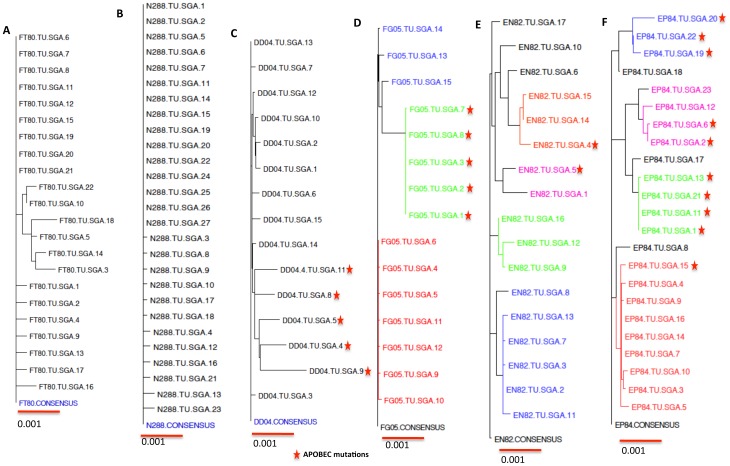
Phylogenetic analysis of full-length env sequences from VSV-HA (Group III) macaques. The full-length envelope sequences obtained from al the control animals infected with SIVsmE660 were compared against the respective consensus sequence and were phylogenetically analyzed (A) FT80 (B) N288 (C) DD04 (D) FG05 (E) EN82 and (F) EP84. Multiple lineages of T/F viruses infecting the same animal were shown in different colors.

This group of animals was primed and boosted twice with VSV expressing influenza HA. All the macaques in this group were infected after IR challenge with SIVsmE660. Three out of six macaques showed persistently high PVLs (EN82, FG05 and N288) (Fig. 1) whereas the remaining 3 exhibited relative control of SIVe660 with a decline in viral load after the peak viremia (FT80, DD04 and EP84). SGA was performed on the plasma samples of all control animals at peak viremia and phylogenetic trees were constructed. (Fig. 7). The variant lineages infecting the challenged macaques are depicted in different colors. In case of FT80 and N288 animals, we analyzed 22 and 26 full-length envelope sequences respectively. Only a single founder virus lineage was infecting these animals based on the highlighter plot analysis (Fig. 7A, B). In case of DD04, among 15 envelopes sequenced, several APOBEC mutations were present and after their elimination this animal was infected with only one founder virus (Fig. 7C). We sequenced 15 envelopes from FG05, noted 3 different founder virus lineages while EN82 was infected with at least 3 founder virus envelope sequences (Fig. 7D, E). We obtained 23 full-length envelope sequences from EP84 and detected more than 4 founder viruses from this animal (Fig. 7F). We also performed the Poisson-Fitter analysis on all the envelope sequences obtained from each animal (data not shown). The envelope sequences from N288, FT80 and DD04 all fit the Poisson distribution, since all of them were infected with a single founder virus that evolved randomly to exhibit star like phylogeny. Since the macaques FG05, EN82 and EP84 were infected with multiple viruses, their envelope sequences failed to follow Poisson-Fitter distribution.

In the case of gag-env-GM-CSF and HA immunized groups, half of the macaques were infected with multiple founder viruses and the other half with a single founder virus (Table 2). This is in contrast to the data from gag-env group, where 2 of 2 of macaques were infected with a single founder virus. The significance of this difference, if any, was not be established because of the low number of infected macaques in the gag-env vaccinated group. Further studies are needed with additional animals for sequencing of the entire SIV genome of the founder viruses from infected animals.

**Table 2:** Summary of SGA analysis of envelope sequences.

Group*	Prime (Day 0) (i.m. and i.n)	Boost (Day 49) (i.m)	Boost (Day 112) (i.m. and i.n)	Virus Challenge (Day 147) (4000TCID_50_ i.r)
I	VSV (NJ)	SFV-G replicon	VSVG (I)	SIVsmE660
	SIV Gag, Env	SIV Gag, Env	SIV Gag, Env	
II	VSVG (NJ)	SFV-G replicon	VSVG (I)	SIVsmE660
	SIV, Gag, Env, GM-CSF	SIV Gag, Env	SIV Gag, Env	
III	VSVG (NJ)	SFV-G replicon	VSVG (I)	
	Influenza HA	Influenza HA	Influenza HA	SIVsmE660

### Comparison of founder virus envelope sequences to E660 stock and between different groups of infected RhM

**Figure 8:**
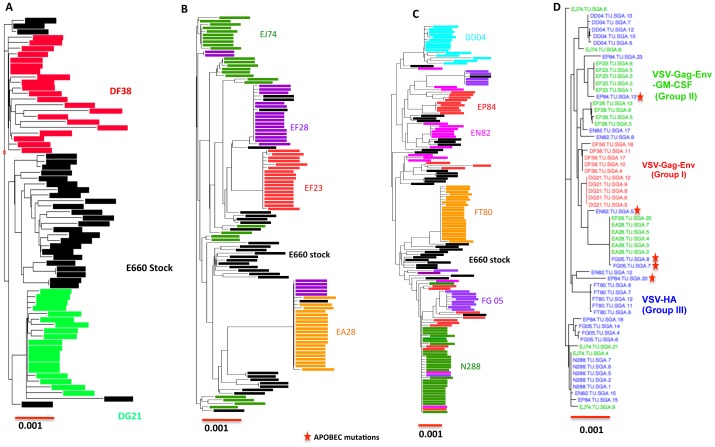
Comparison of T/F env sequences to SIVsmE660 stock sequence and to T/F env sequences from different infected macaques. We phylogenetically compared the T/F env sequences obtained from all the infected macaques to the sequences obtained from challenge virus from groups (A) Gag-Env (B) Gag-Env-GM-CSF and (C) VSV-HA macaques. (D) Representative env sequences (5) from each infected macaque from each lineage of T/F viruses from all of the infected animals were compared to each other.

We phylogenetically analyzed the combined full-length envelope sequences obtained from all SIVsmE660 infected animals. The envelope sequences from different founder viruses had overlap with challenge stock virus sequence even though they were not identical to the stock envs (Fig. 8A). The two founder lineages from the gag-env immunized (Group I) animals were separated into two different regions of the tree with none of the envelope sequences overlapping with gag-env-GM-CSF (Group II) or control HA (Group III). In the case of gag-env-GM-CSF macaques, full-length envelope sequences from EF28, EA28 overlapped demonstrating that there was common founder virus envelope infecting both the RhM. Furthermore; sequences from EF28 and EJ74 were found in multiple sites on the phylogenetic tree confirming that these animals were infected with more than one lineage. Sequences from the control HA vaccinated animals DD04, FT80 and N288 showed evidence of a single founder variant each whereas sequences from EN82, EP84 and FG05 exhibited multiple lineages. The sequences from EJ74 were the most variable of all the envelope sequences and were distributed along the entire phylogenetic tree. Interestingly, a few of the envelope sequences from FG05, EF28 and EA28 were identical suggesting a potential fitness advantage for this particular linage. In VSV-HA immunized animals many T/F sequences were identical in several animals (N288, EP84, EN82, FG05 and EJ74) again suggesting a selective advantage to this particular lineage in transmission. Finally, we analyzed 5 T/F envelope sequences (representing majority lineages) from all the infected macaques and compared them phylogenetically (Fig. 8D). We observed that there is not only an overlap of envelope sequences between different animals of the same immunized group but also between different groups of macaques. The T/F lineage from both the gag-env group infected macaques grouped separately and didn't overlap with any other lineages. The envelope sequences from gag-env-GM-CSF and HA RhM did overlap with each other. The T/F viral envelope sequences from all the infected macaques will be deposited in the Genbank. We further analyzed the glyocsylation status of founder viruses by using the N-Glycosite software from HIV database web site (http://www.hiv.lanl.gov/). HIV contains two heavily glycosylated proteins gp120 and gp41, which mediate attachment of virions to CD4 on the cell surface. Further, protein N-glycosylation is critical to the pathogenesis of HIV at the levels of viral infectivity and cytopathicity but not at the level of virus replication or host-cell infectability (38–40). We wanted to examine if there were any differences in the glycosylation profiles of envelopes derived from gag-env vaccinated macaques to those in the control HA group. We failed to observe any differences in the glycosylation profiles between these two groups and on an average each sequence had 25–27 N-glycosylation sites (data not shown).

We also compared the consensus amino acid sequences of e660 stock to consensus sequences of single founder lineages from gag-env, gag-env- GM-CSF and HA vaccinated groups. All the single T/F lineages from different vaccinated macaques differed from the consensus e660 stock envelope sequence in the cytoplasmic tail (amino acids 878-885) of the envelope.

In conclusion, we characterized the SIVsmE660 viral stock used for challenging the rhesus macaques. 58 full-length envelope sequences were analyzed and the stock had a diversity of 1.4%. Further, we also evaluated the 246 full-length envelope sequences obtained from three different groups of rhesus macaques that were immunized with various VSV constructs. We observed that the rhesus immunized with VSV expressing SIV gag and env showed only one founder virus as compared to other immunized groups which had animals infected with multiple founder viruses. Future studies involve deciphering the functional role of these envelope sequences for characterizing their susceptibility to antibody-mediated neutralization that could elucidate the role of the antibody. Additionally, it is also likely that other genes/proteins of SIV/HIV play an important role in correlating the vaccine efficacy to protection. Therefore, in future studies we will perform SGA of the whole genome to better understand their role in vaccine mediated protection.
